# Recurrent Clostridioides difficile Infection (CDI) in Patients Treated With Vancomycin at Johns Hopkins Aramco Healthcare (JHAH), Dhahran, Saudi Arabia

**DOI:** 10.7759/cureus.85116

**Published:** 2025-05-31

**Authors:** Hashim H Alnahwi, Rawan J AlGhawi, Hassan Ahmed A. Alsahaf, Elwaleed Ahmed

**Affiliations:** 1 Internal Medicine, Johns Hopkins Aramco Healthcare, Dhahran, SAU; 2 Infectious Disease, Johns Hopkins Medicine International, Dhahran, SAU

**Keywords:** clostridioides difficile recurrence rate, clostridium difficile, clostridium difficile treatment, dhahran, johns hopkins aramco healthcare, recurrent clostridioides difficile infection, saudi arabia, vancomycin, vancomycin treatment

## Abstract

Introduction

*Clostridioides difficile *infection (CDI) is a leading cause of healthcare-associated diarrhea with a significant risk of recurrence, posing challenges for patient management and infection control. Identifying risk factors for recurrence is essential to improve outcomes and prevent relapses.

Methods

This retrospective cohort study included 860 adult patients (≥18 years) treated with vancomycin for CDI at Johns Hopkins Aramco Healthcare (JHAH) in Dhahran, Saudi Arabia, between January 2015 and December 2020. Patients with confirmed CDI based on stool polymerase chain reaction (PCR) or toxin assays, complete medical records, and adequate follow-up data were included. The study excluded those not treated with vancomycin, under 18 years of age, with incomplete records, those who received fecal microbiota transplantation or experimental treatments, and those lacking follow-up data. Data on demographics, comorbidities, hospitalization, medication use, and recurrence were analyzed using univariate and multivariate logistic regression models.

Results

Univariate analysis showed that age 40-65 years (OR = 1.53; 95% CI: 1.024-2.285; p = 0.038), age >65 years (OR = 1.894; 95% CI: 1.282-2.799; p = 0.001), cirrhosis (OR = 9.104; 95% CI: 1.233-67.192; p = 0.03), hospitalization (OR = 1.974; 95% CI: 1.417-2.749; p < 0.0001), and type 2 diabetes mellitus (OR = 1.65; 95% CI: 1.106-2.462; p = 0.014) were significantly associated with CDI recurrence. After adjusting for confounders, only hospitalization remained a statistically significant independent predictor (OR = 1.597; 95% CI: 1.098-2.325; p = 0.014).

Conclusion

Hospitalization was identified as the most significant independent risk factor for CDI recurrence. These findings highlight the need for enhanced infection control practices and close monitoring of hospitalized patients with CDI. Future prospective and multicenter studies are recommended to validate these results and explore additional modifiable risk factors to reduce recurrence rates.

## Introduction

*Clostridioides difficile *infections (CDIs), caused by the Gram-positive, spore-forming bacterium *C. difficile* (CD), represent a major challenge in clinical settings due to their potential to cause severe gastrointestinal conditions [1,2], including mild diarrhea, pseudomembranous colitis, and toxic megacolon. The pathogen's virulence is attributed to its production of toxins A and B, which disrupt the colonic epithelial barrier and induce inflammatory responses [3]. CDI prevalence varies widely across regions. In Asia, hospital-based rates range from 2.6 to 25.2 cases per 10,000 patient days, with mortality around 9%. South America reports rates between 8.3% and 41% [4], while incidence can reach 12.9 per 1,000 admissions [5]. In the United States, about 101 cases occur per 100,000 people annually, with nearly equal distribution between community and healthcare-associated infections. Around 250,000 people are infected each year, resulting in about 14,000 deaths. Globally, CDI remains a significant public health issue. A review also showed average hospital rates at 2.24 per 1,000 admissions and 3.54 per 10,000 patient days, with community rates at 14.34 per 100,000, the highest in North America [6]. The CD is mainly spread via the fecal-oral route, with antibiotic use being the most significant risk factor due to its disruption of gut microbiota. Treatment typically involves antibiotics such as metronidazole, fidaxomicin, and vancomycin [7]. Other medications, such as proton pump inhibitors (PPIs) and histamine-2 receptor antagonists (H2RAs), also raise the risk by reducing gastric acidity and weakening immune defenses [8,9]. Additional common risk factors include older age, underlying health conditions, prolonged hospitalization, residence in long-term care facilities, and exposure to healthcare environments [8,10]. Despite the efficacy of vancomycin, an antibiotic commonly used to treat CDI, recurrence rates remain troublingly high, estimated between 20% and 30% [11]. The rise of vancomycin-resistant strains and underlying conditions like cirrhosis, immunosuppression, and gut disorders weaken immunity and disrupt gut microbiota, increasing CDI recurrence. Standard treatments such as metronidazole and vancomycin have limited success in preventing relapses, leading to new therapies like microbiome-based SER-109 [12]. This recurrence is often driven by factors such as the bacterium's ability to form highly resistant spores, which can persist on surfaces and within the gastrointestinal tract, leading to re-infection [13]. Understanding these dynamics is critical for developing more effective treatment protocols and implementing targeted infection control strategies to mitigate the burden of CDI in healthcare settings.

This study investigates factors linked to recurrent CDI in patients treated with vancomycin at Johns Hopkins Aramco Healthcare (JHAH), Dhahran, Saudi Arabia. By examining clinical and treatment variables, it aims to improve outcomes through targeted interventions. Identifying key risk factors will support personalized care, enhance infection control, and guide future preventive strategies.

## Materials and methods

Study design

This retrospective cohort study with 860 patients aimed to identify factors contributing to recurrent CDI in adult patients (aged 18 years and older) treated with vancomycin at JHAH in Dhahran between January 2015 and December 2020. All eligible patients meeting the inclusion criteria within this period were systematically included to minimize selection bias. Inclusion criteria required a confirmed CDI diagnosis through stool polymerase chain reaction (PCR) testing or toxin assays, complete electronic medical records, and sufficient follow-up data to assess CDI recurrence. The study involved a comprehensive review of patient records to analyze patterns, trends, and outcomes related to CDI recurrence, focusing on key variables such as underlying medical conditions, treatment adherence, and other risk factors. Exclusion criteria included patients not treated with vancomycin, those with incomplete medical records, individuals under 18 years of age, those who received fecal microbiota transplantation or experimental therapies, and patients diagnosed outside the study timeframe or lacking adequate follow-up for recurrence assessment.

The research protocol was rigorously reviewed and approved by the JHAH Institutional Review Board (IRB), ensuring compliance with ethical and regulatory standards. This approval was essential for maintaining the integrity of the study, safeguarding patient confidentiality, and ethically managing sensitive medical data. The study adhered to established research guidelines, prioritizing informed consent, privacy protection, and ethical treatment of human subjects. Through this rigorous approach, the study aimed to generate reliable and credible findings that could contribute to improved patient care and infection control measures in healthcare settings.

Data collection

Data for this study were carefully extracted from electronic medical records, compiling a comprehensive dataset covering patient demographics, clinical history, treatment details, and CDI outcomes. The diagnosis was established using stool tests, with 62.91% of cases identified by PCR for CD and by CD toxin assay. Key variables included age, gender, and underlying medical conditions that could impact recurrence risk. Specifically, data on liver cirrhosis, Crohn's disease, and ulcerative colitis were collected due to their potential influence on immune response and gut health. A history of cancer chemotherapy was noted for its effects on immunity and microbiome, while hypertension and type 2 diabetes were recorded for their broader impact on infection outcomes. The use of PPIs, which may alter gastric acidity and affect CDI risk, was also documented. Additionally, hospitalization status was included to assess its role in increasing exposure to healthcare-associated infections. This detailed dataset aimed to identify risk factors for CDI recurrence and support the development of targeted prevention strategies.

Statistical analysis

The Shapiro-Wilk test was used to test the normality across continuous variables. Descriptive statistics were used to summarize the study sample, providing insights into demographics and clinical characteristics. Continuous variables, like age, were reported using mean and standard deviation (SD) or median and range for non-normally distributed variables, while categorical variables, such as gender and medical history, were summarized using frequencies and percentages. Variables with more than 20% missingness were excluded from the final analysis.

For inferential analysis, chi-square and Fisher's exact test assessed associations between recurrent CDI and categorical variables, such as cirrhosis and hospitalization. Welch’s t-test compared the mean age between recurrent and non-recurrent cases. The Mann-Whitney test was used to investigate a comparison between CDI recurrence and no CDI recurrence in age using median and range. Odds ratios (ORs) with 95% confidence intervals (CI) quantified the strength of associations between risk factors and CDI recurrence using binary logistic regression. Also, multivariate logistic regression was conducted to adjust for different confounders. Adjustments for multiple comparisons were made using Bonferroni correction to reduce the risk of false positives. These methods helped identify key predictors, informing targeted prevention and treatment strategies. All statistical analyses were performed using R, a powerful software for statistical computing and data visualization.

## Results

Patient characteristics

The patient population had a mean age of 53.77 years (SD = 27.65), indicating a wide age range affected by CDI. Females represented 56.98% (n = 490), and males 43.02% (n = 370), showing a slight female predominance. The underlying gastrointestinal or liver conditions were uncommon, with cirrhosis in 3.60% (n = 31), Crohn’s disease in 1.51% (n = 13), and ulcerative colitis in 2.21% (n = 19). Treatment with fidaxomicin was rare, used in only 0.47% (n = 4) of cases.

Diagnostic testing favored stool PCR in 62.91% (n = 541) of patients, while 37.09% (n = 319) were diagnosed by toxin assay, reflecting a preference for the more sensitive PCR method.

**Figure 1:**
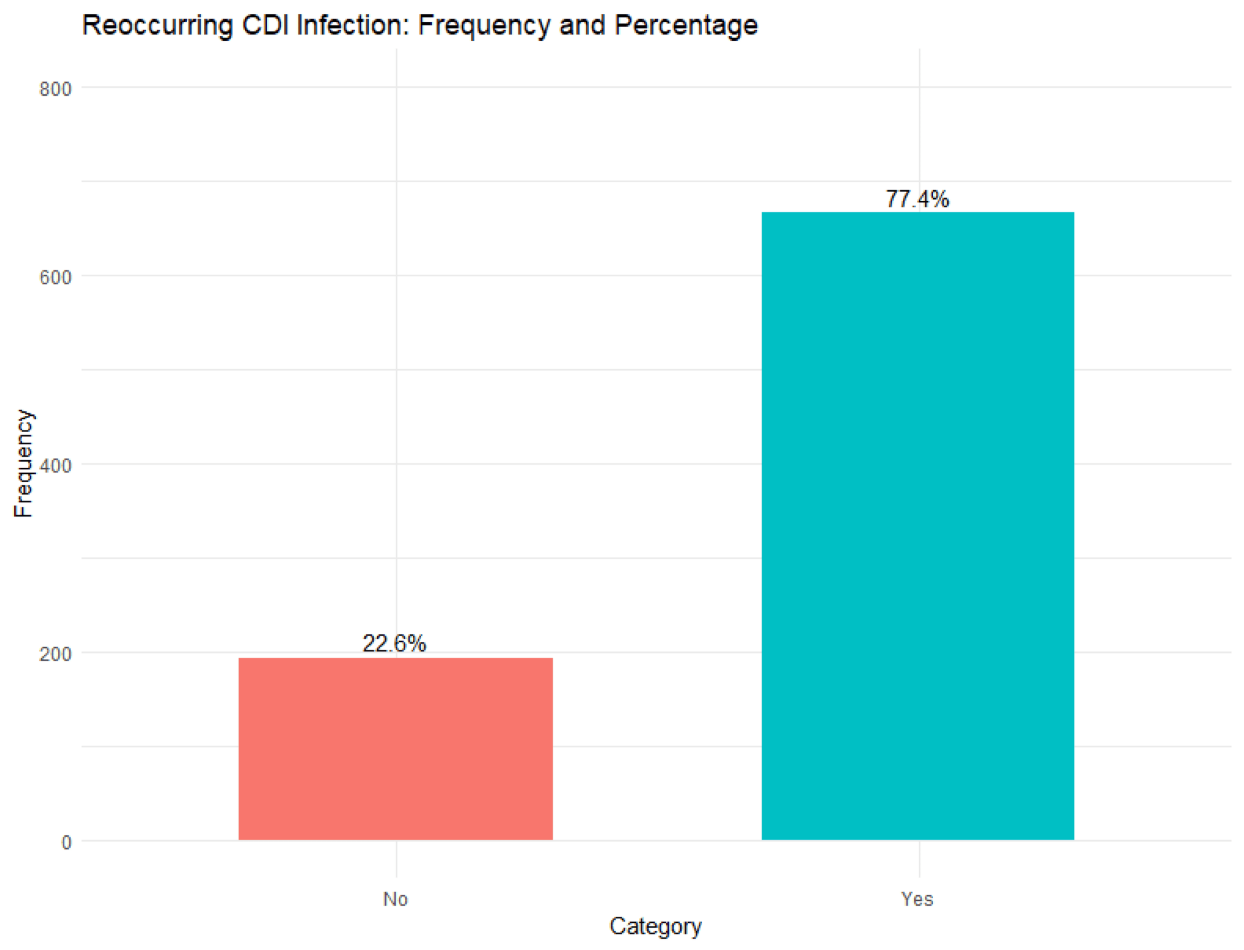
Recurrence rate of CDI CDI: *Clostridioides difficile* infection

The clinical burden was substantial: 69.07% (n = 594) were hospitalized, and a high rate of recurrent CDI infections (77.44%, n = 666) underscored the relapsing nature of the disease (Figure 1). Most patients (63.72%, n = 548) were on PPIs, which are known to increase CDI risk. Additionally, notable comorbidities included cancer-chemotherapy history (20.81%, n = 179), hypertension (30.58%, n = 263), and type 2 diabetes mellitus (T2DM) (25.35%, n = 218), all of which may impact CDI outcomes and management (Table 1).

**Table 1:** Demographic and clinical characteristics of the study sample (n = 860) PPI: proton pump inhibitor; PCR: polymerase chain reaction; CDI: *Clostridioides difficile* infection

Variable	n (proportion)
Age, mean (SD)	53.77 ± 27.65
Gender	
Female (%)	490 (56.98)
Male (%)	370 (43.02)
Medical history	
History of cirrhosis (%)	31 (3.60)
History of Crohn’s disease (%)	13 (1.51)
History of ulcerative colitis (%)	19 (2.21)
Treated with fidaxomicin (%)	4 (0.47)
Test type	
Stool, *Clostridioides difficile* by PCR (%)	541 (62.91)
Stool, *Clostridium difficile* toxin assay (%)	319 (37.09)
Clinical characteristics	
Hospitalized (%)	594 (69.07)
Recurrent CDI infection (%)	666 (77.44)
Patient on PPI (%)	548 (63.72)
Cancer-chemotherapy history (%)	179 (20.81)
Hypertension (%)	263 (30.58)
Type 2 diabetes mellitus (%)	218 (25.35)

Risk factors and associations in recurrent CDI

Fisher's exact and chi-square tests, used to evaluate the association between categorical variables, revealed significant differences in the probability of recurrent CDI for several key factors. There was a significant association between CDI recurrence and age categories, as the risk of recurrence increased with increasing age (p = 0.005). The analysis demonstrated that patients with a history of cirrhosis had a significantly higher probability of experiencing recurrent CDI, with a p-value of 0.007. This finding indicates a robust association between liver cirrhosis and an increased risk of CDI recurrence, suggesting that patients with compromised liver function may have a heightened susceptibility to persistent infections.

Similarly, the data showed that patients who had been hospitalized exhibited a significantly higher probability of recurrent CDI, with a p-value of less than 0.0001. This result underscores the substantial impact of hospitalization on the likelihood of CDI recurrence, likely due to factors such as increased exposure to healthcare-associated pathogens and antibiotic use during hospital stays. There was a statistically significant association between T2DM and recurrent CDI infection (p = 0.0146), with higher recurrence observed among diabetic patients. The association between a history of ulcerative colitis and a higher risk of recurrent CDI is not significant, with a p-value of 0.0925.

In contrast, the analysis found no significant differences in the probability of recurrent CDI associated with several other factors. The presence of Crohn's disease did not show a statistically significant impact on the likelihood of CDI recurrence, indicating that this condition may not be a major risk factor in this context. Additionally, the use of PPIs did not demonstrate a significant association with CDI recurrence, indicating that PPI use may not significantly influence the likelihood of recurrent infections in this study. No significant association was observed in the diagnostic parameters (toxin assay and PCR) for CDI recurrence (p = 0.179).

These findings highlight key factors associated with CDI recurrence and suggest areas for further research to better understand and manage the risk of recurrent CDI (Table 2).

**Table 2:** Chi-square and Fisher exact test of factors associated with recurrent CDI PPI: proton pump inhibitor; PCR: polymerase chain reaction; CDI: *Clostridioides difficile *infection

Variable	Yes (n = 666) (%)	No (n = 194) (%)	p-value
Gender			
Female	382 (77.96)	108 (22.04)	0.6812
Male	284 (76.76)	86 (23.24)	
Age category			
<40	166 (70.3)	70 (29.7)	0.005
40-65	214 (78.4)	59 (21.6)	
>65	283 (81.8)	63 (18.2)	
History of cirrhosis			
No	636 (76.72)	193 (23.28)	0.007
Yes	30 (96.77)	1 (3.23)	
History of Crohn’s disease			
No	655 (77.33)	192 (22.67)	0.7433
Yes	11 (84.62)	2 (15.38)	
History of ulcerative colitis			
No	648 (77.05)	193 (22.95)	0.0925
Yes	18 (94.74)	1 (5.26)	
Hospitalized			
No	183 (68.80)	83 (31.20)	<0.0001
Yes	483 (81.31)	111 (18.69)	
Patient on PPI			
No	233 (74.68)	79 (25.32)	0.1498
Yes	433 (79.01)	115 (20.99)	
Cancer-chemotherapy history			
No	525 (77.09)	156 (22.91)	0.6882
Yes	141 (78.77)	38 (21.23)	
Hypertension			
No	453 (75.88)	144 (24.12)	0.111
Yes	213 (80.99)	50 (19.01)	
Type 2 diabetes mellitus			
No	484 (75.39)	158 (24.61)	0.0146
Yes	182 (83.49)	36 (16.51)	
Diagnosis			
Toxin assay	255 (79.9)	64 (20.1)	0.179
PCR	411 (75.9)	130 (24.1)	

Univariate analysis of risk factors for CDI recurrence

According to univariate logistic regression, several factors were significantly associated with an increased risk of recurrent CDI. Age was a strong predictor. Individuals aged 40-65 years had 1.53 times higher odds of recurrence (95% CI: 1.024-2.285, p = 0.038), and those older than 65 years had 1.894 times higher odds (95% CI: 1.282-2.799, p = 0.001), compared to patients under 40. A history of cirrhosis was also strongly associated with recurrence, showing an OR of 9.104 (95% CI: 1.233-67.192, p = 0.03). Additionally, hospitalization (OR = 1.974; 95% CI: 1.417-2.749; p < 0.0001) and T2DM (OR = 1.65; 95% CI: 1.106-2.462; p = 0.014) were significant predictors of recurrence (Table 3).

**Table 3:** Binary logistic regression OR: odds ratio; PPI: proton pump inhibitor; PCR: polymerase chain reaction * indicates a p-value of <0.05 is statistically significant

Parameter	OR (95%CI)	p-value
Gender		
Male	1	-
Female	1.071 (0.776, 1.478)	0.676
Age category		
<40	1	-
40-65	1.53 (1.024, 2.285)	0.038*
>65	1.894 (1.282, 2.799)	0.001*
History of cirrhosis	9.104 (1.233, 67.192)	0.03*
History of ulcerative colitis	5.361 (0.711, 40.417)	0.103
History of Crohn’s disease	1.612 (0.354, 7.336)	0.537
Hospitalization	1.974 (1.417, 2.749)	<0.0001*
PPI use	1.277 (0.92, 1.772)	0.144
Chemotherapy history	1.103 (0.739, 1.645)	0.633
Hypertension	1.354 (0.944, 1.942)	0.099
Diabetes type 2	1.65 (1.106, 2.462)	0.014*
Test		
Toxin assay	1	-
PCR	0.793 (0.566, 1.112)	0.179

Multivariate analysis of risk factors for CDI recurrence

After adjusting for potential confounding variables in multivariate logistic regression, most associations observed in the univariate analysis lost statistical significance. The only factor that remained independently associated with CDI recurrence was hospitalization, with an adjusted OR of 1.597 (95% CI: 1.098-2.325, p = 0.014). This suggests that while factors such as age, cirrhosis, and diabetes may contribute to the risk, hospitalization itself may play a more direct and consistent role in predicting CDI recurrence in this patient population (Table 4).

**Table 4:** Multivariate logistic regression OR: odds ratio; PPI: proton pump inhibitor; PCR: polymerase chain reaction * indicates a p-value of <0.05 is statistically significant

Parameter	OR (95% CI)	p-value
Gender		
Male	1	-
Female	1.131 (0.809, 1.582)	0.471
Age category		
<40	1	-
40-65	1.331 (0.823, 2.153)	0.244
>65	1.549 (0.939, 2.556)	0.087
History of cirrhosis	5.111 (0.667, 39.168)	0.116
History of ulcerative colitis	4.938 (0.605, 40.336)	0.136
History of Crohn’s disease	2.267 (0.483, 10.641)	0.3
Hospitalization	1.597 (1.098, 2.325)	0.014*
PPI use	0.969 (0.67, 1.403)	0.868
Chemotherapy history	0.94 (0.603, 1.466)	0.785
Hypertension	1.031 (0.684, 1.555)	0.884
Diabetes type 2	1.316 (0.847, 2.044)	0.222
Test		
Toxin assay	1	-
PCR	0.775 (0.545, 1.102)	0.156

## Discussion

This study highlights several potential risk factors for CDI recurrence. Univariate analysis revealed that older age, a history of cirrhosis, hospitalization, and T2DM were significantly associated with increased risk of recurrence. However, after adjusting for confounding variables, only hospitalization remained an independent predictor. These findings emphasize the importance of hospital-based factors in CDI recurrence and suggest that preventive strategies should prioritize infection control measures and tailored management of hospitalized patients to reduce the risk of relapse.

These results are of clinical importance for treatment and prevention and have value in providing information about risk factors promoting the recurrence of CDI. One significant finding was the association between older age and an increased likelihood of CDI recurrence (p = 0.015), which is consistent with previous research suggesting that aging-related immune decline and a higher prevalence of comorbidities contribute to greater susceptibility [14]. Age was a key determinant; patients aged 40-65 years had significantly higher odds of recurrence compared to those under 40 (OR = 1.53, 95% CI: 1.024-2.285, p = 0.038), and the risk was even greater among those over 65 years (OR = 1.894, 95% CI: 1.282-2.799, p = 0.001).

Another interesting finding of this study is the very high correlation between recurrent CDI and hospitalization (p < 0.0001). When controlling for confounding variables through multivariate logistic regression, only hospitalization remained a statistically significant independent predictor of CDI recurrence (OR = 1.597, 95% CI: 1.098-2.325, p = 0.014). This finding supports earlier studies that have shown that nosocomial settings promote the spread of CD mainly because of environmental contamination, increased patient susceptibility, and the extensive use of antibiotics, as supported by this systematic review and meta-analysis that hospital environmental surfaces and medical devices are significant sources of CDI [15]. Hospitalized patients have widespread antibiotic treatment, which upsets the normal gut flora and exposes them to CDI [16]. Additionally, the actual hospital setting acts as a reservoir for CD spores that enhance the risk for reinfection, as found here in medical personnel's clothes, which can act as reservoirs for the spores [17]. Environmental decontamination, hand hygiene, and antibiotic stewardship programs are just some of the stringent infection control measures that are necessary to reduce the risk for recurrent CDI in healthcare settings [18].

This study's connection between cirrhosis and recurrent CDI is another important finding (p= 0.051). After univariate analysis, a history of cirrhosis was strongly associated with recurrence, with an OD of 9.104 (95% CI: 1.233-67.192, p = 0.03), suggesting that liver dysfunction may impair host immunity or alter gut microbiota, promoting CDI relapse. Immune dysregulation and changes in gut microbiota are common in cirrhosis patients [19], and they may make them more vulnerable to CDI recurrence [20]. Cirrhosis patients have a higher chance of recurrence because their immune systems are less able to effectively fight off infections [21]. Furthermore, the disturbance of intestinal homeostasis in cirrhosis, which is marked by an increase in intestinal permeability and bacterial proliferation, might foster CD colonization and tenacity, as meta-analysis indicates the increase in small intestinal bacterial growth in patients with nonalcoholic liver disease, which leads to cirrhosis and contributes to CD colonization [22]. According to the study's findings, cirrhotic patients are particularly susceptible to CDI, and specific interventions like probiotic therapy and gut microbiota modification may help lower the risk of recurrence in this high-risk population [23]. In another study, the probiotics have a beneficial role in immune function and can improve liver function in stable cirrhosis, but their effect on gut barrier and bacterial translocation in cirrhotic patients is minimal, as explained here in a randomized clinical trial [24].

It is also noteworthy that this study found a correlation between T2DM and recurrent CDI (p = 0.014) through Fisher's test. After univariate analysis, among chronic comorbidities, T2DM was significantly associated with recurrence (OR = 1.65, 95% CI: 1.106-2.462, p = 0.014), potentially reflecting immune dysregulation or increased antibiotic exposure in diabetic patients. Immune dysfunction, chronic inflammation, and metabolic dysregulation are all hallmarks of diabetes that can raise the risk of infection [25]. Diabetes patients are more prone to bacterial infections, including CDI, because they frequently have delayed immune responses and compromised neutrophil function [26]. Further raising the risk of recurring infections, hyperglycemia has also been demonstrated to encourage bacterial growth and compromise the integrity of the intestinal epithelial barrier [27]. According to the study's findings, to reduce the risk of recurrence, clinicians must closely monitor diabetic patients with CDI and take into account customized treatment strategies like glycemic control optimization and adjunctive therapies [28].

Likewise, there is ample evidence in the literature linking cirrhosis to CDI recurrence, with research emphasizing the part that immunological impairment and gut dysbiosis play in heightened vulnerability to infection. PPI use and CDI recurrence did not significantly correlate in this study (p = 0.1498), which is in contrast to some earlier findings that suggested PPIs may raise the risk of CDI by changing stomach acidity and encouraging bacterial growth [29,30]. When controlling for confounding variables through multivariate logistic regression, only hospitalization remained a statistically significant independent predictor of CDI recurrence (OR = 1.597, 95% CI: 1.098-2.325, p = 0.014). While age over 65 years and a history of cirrhosis retained elevated ORs, they were no longer statistically significant (p = 0.087 and p = 0.116, respectively). Similarly, T2DM and other chronic conditions such as hypertension, chemotherapy history, and PPI use did not maintain statistical significance after adjustment. These findings highlight the importance of hospitalization as a primary driver of recurrence risk, possibly due to increased exposure to healthcare-associated pathogens, antibiotic use, or overall patient frailty. The absence of significance in other variables in the multivariate model underscores the multifactorial nature of CDI recurrence and suggests that hospitalization may encapsulate a range of overlapping risks.

The analysis identified several significant associations in the univariate regression, where older age (particularly ≥40 years), history of cirrhosis, hospitalization, and T2DM were all linked to a higher risk of CDI recurrence. However, following multivariate adjustment, hospitalization remained a statistically significant independent risk factor. This finding underscores the importance of inpatient care dynamics in the recurrence of CDI and suggests that other variables may act as confounders when not controlled for. These insights highlight the complexity of CDI recurrence and the necessity for multifactorial prevention approaches.

Limitations

This study has several limitations that should be considered when interpreting the findings. The retrospective design may introduce selection and restrict the ability to establish causality between the identified risk factors and CDI recurrence. Furthermore, unmeasured confounders, such as variations in antibiotic resistance patterns and patient adherence to treatment, were not accounted for in the analysis, potentially influencing the study’s conclusions. Besides that, it is possible that the results of this study will not be valid to the entire extent for populations outside the particular healthcare settings in which the data were collected. Patient populations, medical treatment, and environmental factors may affect the degree to which the outcomes can be generalized. Another limitation is the limited sample of patients treated with fidaxomicin, and thus it is difficult to evaluate precisely how the recurrence rates are affected by it. To overcome such limitations and reveal a more complete view of the risk factors of CDI recurrence in the future, larger sample studies as well as greater numbers of patients should be performed.

Recommendations for future research

Future research should focus on addressing the limitations of this study and further exploring factors contributing to recurrent CDI. Prospective, multicenter studies are needed to confirm the associations identified and enhance the generalizability of findings across diverse healthcare settings. Investigating the role of antibiotic resistance patterns and patient adherence to treatment could provide a more comprehensive understanding of CDI recurrence. Additionally, studies evaluating the effectiveness of alternative treatment strategies, such as fidaxomicin, fecal microbiota transplantation, and immunotherapies, may offer valuable insights into reducing recurrence rates. Finally, research on predictive models incorporating clinical, microbiological, and genetic factors could aid in early risk stratification and personalized management of high-risk patients. Such future research can help to create improved CDI prevention and treatment interventions and enhance patient outcomes by closing these gaps in knowledge.

## Conclusions

The findings of this study highlight that several factors were associated with an increased risk of CDI recurrence, including older age, history of cirrhosis, hospitalization, and T2DM. However, after accounting for other variables, only hospitalization remained a significant predictor of recurrence. This suggests that hospital-related factors play a crucial role in the relapse of CDI and should be a primary focus in prevention efforts. Emphasizing infection control protocols and individualized care for hospitalized patients may help reduce recurrence rates.
